# Editorial: Beneficial and detrimental host cellular responses against *Mycobacterium tuberculosis* infection

**DOI:** 10.3389/fcimb.2023.1332084

**Published:** 2023-11-27

**Authors:** Joaquin Miguel Pellegrini, María Paula Morelli, María Isabel Colombo, Verónica Edith García

**Affiliations:** ^1^Centre d’Immunologie de Marseille Luminy, INSERM, Centre national de la recherche scientifique (CNRS), Aix-Marseille Université, Parc Scientifique et Technologique de Luminy, Case 906, Marseille, France; ^2^Departamento de Química Biológica, Facultad de Ciencias Exactas y Naturales, Universidad de Buenos Aires, Ciudad Autónoma de Buenos Aires, Argentina; ^3^Instituto de Química Biológica de la Facultad de Ciencias Exactas y Naturales (IQUIBICEN), Facultad de Ciencias Exactas y Naturales, Universidad de Buenos Aires, Consejo Nacional de Investigaciones Científicas y Técnicas (CONICET), Ciudad Autónoma de Buenos Aires, Argentina; ^4^Instituto de Histología y Embriología de Mendoza, Facultad de Ciencias Médicas, Universidad Nacional de Cuyo-CONICET, Mendoza, Argentina

**Keywords:** tuberculosis, host-pathogen interactions, *Mycobacterium tuberculosis*, immune response, immunometabolism, genetic

Tuberculosis (TB) is a contagious infectious disease caused by *Mycobacterium tuberculosis* (*Mtb*), an exceptionally successful pathogen. In fact, it is estimated that *Mtb* has killed nearly 1000 million people since the XIX century. The most updated mortality data indicate that in 2021, 1.4 million people died of TB (World Health Organization, 2022), converting this pathogenic bacteria into the second leading infectious cause of death globally after COVID-19. *Mtb*, a microorganism that has produced suffering and death since it was identified almost 150 years ago, urgently requires a better vaccine to prevent TB-dependent morbidity and mortality (Rahlwes et al., 2023). The immune response against *Mtb* is highly complex. *Mtb* invades humans by air and establishes infection in the lung by using a large number of different virulence factors. After infection, *Mtb* interacts with different cells of the innate and adaptive immune compartments. These cells play an important role in the modulation and development of the pathology. *Mtb*-infected alveolar macrophages and dendritic cells are both reservoirs of infection and function to activate an adaptive immune response. *Mtb*-infected cells migrate from the lung to draining lymph nodes to prime and activate T and B cells to limit progression of infection (Urdahl et al., 2011; Shaler et al., 2012; Carpenter and Lu, 2022). Infected macrophages, along with non-infected phagocytes, (macrophages, monocytes and neutrophils), and T cells recruited by the inflammation and tissue damage will in turn form the characteristic TB granuloma (Cohen et al., 2022; Cronan, 2022). A well-formed granuloma prevents the progression of infection, limiting tissue damage to a small region, which is beneficial for the host. Most *Mtb*-infected people will contain TB infection at this step and be asymptomatic (latent TB). In the case of the bacteria, the granuloma maintains the pathogen in a state of dormancy avoiding clearance by the immune system (Park et al., 2003). An efficient host protection against *Mtb* infection is associated with the induction, activation and proliferation of T helper 1 (Th1) cells (Cooper et al., 1993; Newport et al., 1996; Salgame, 2005). However, IFN-γ alone is not sufficient for the complete eradication of the bacteria, suggesting that other cytokines might be required for pathogen removal. Accordingly, cellular responses to *Mtb* induce IL-17 production, contributing to granuloma formation and control of bacterial growth (Cooper, 2010). However, excessive IL-17 levels exacerbate inflammation, increasing neutrophil recruitment and tissue damage (Lázár-Molnár et al., 2010). Despite the great steps made in the characterization of the acquired cellular response in TB patients, it remains to be elucidated what exactly constitutes a protective response or leads to disease pathology. Furthermore, how *Mtb* is able to evade host immune surveillance and persist is not fully clarified yet.

Our Research Topic was launched on May 18^th^ 2022 with a planned close on November 11th 2022, but it was extended until October 25^th^ 2023 due to the flow of submissions.

We are pleased to present a comprehensive Research Topic that delves into the intricate web of host-pathogen interactions during TB. Our Research Topic encompasses articles within a diverse range of disciplines, including cellular biology, endocrinology, host and bacterial genetics, epigenetics, immune regulation, immunometabolism, neuroimmunology, and aging. Through these multidisciplinary contributions, we aim to unravel the multifaceted dynamics underlying TB infection, shedding light on the disease’s diverse facets and offering valuable insights for researchers and healthcare professionals. We invite readers to explore the ever-evolving landscape of TB research and foster a deeper understanding of this global health challenge.

## Outline of contributions

In a very complete review, Wang et al. summarized the latest research progress on related signal transduction molecules in the interaction between *Mtb* and the host. In particular, Wang et al. focus on the special *Mtb* cell structure and the research progress of the interaction between this pathogen and host cell surface pattern recognition receptors (such as CLRs, NLRs, and TLRs), as well as macrophage effector molecules and autophagy. The authors reviewed many papers about the fight of the immune system against *Mtb* and the bacteria immune evasion. They even introduced the structure of *Mtb* by describing the particular cell wall of this bacteria, which is mainly composed of peptidoglycan, arabino galactose, trehalose-6,6’-dimycolate, mycolic acid and muramyl dipeptide. In addition they also describe the interaction of *Mtb* effector proteins and the host, and the molecular mechanism of *Mtb* escape from host membrane surface pattern receptors. This study may provide research ideas to find new anti-tuberculosis drugs and the development of host-directed treatment strategies.

There is still much to know about how *Mtb* evades the immune system. Margenat et al., explored the interaction and activity of the *Mtb* virulence factor PtpA, a tyrosine phosphatase released into the macrophage during infection (Bach et al., 2008; Mascarello et al., 2010), on the alpha subunit of the human trifunctional protein enzyme (*h*TFP), a PtpA substrate. During *Mtb* infection, PtpA interacts with several eukaryotic proteins in the macrophage cytosol, modulating phagosome maturation, innate immune response, apoptosis, and potentially host-lipid metabolism. Interestingly, it has been shown that the alpha subunit of *h*TFP is no longer detected in mitochondria during macrophage infection with the virulent *Mtb* H37Rv. Therefore, here, Margenat et al. investigated if PtpA could be the bacterial factor responsible for this effect by analysing the PtpA activity and interaction with *h*TFP_α_. The authors were able to demonstrate that PtpA in fact promotes the survival of mycobacteria in infected cells, affecting not only the innate immunity response (Wang et al., 2017; Bach et al., 2008; Wang et al., 2015; Wang et al., 2020) and apoptosis (Poirier et al., 2014), but also macrophage pathways involved in lipid metabolism.

Ma et al. reported that heme oxygenase-1 (HMOX1) is an essential regulator of *Mtb*-induced ferroptosis. The outcome of the host-pathogen interaction between macrophages and *Mtb* is critical for the development of TB (Xu et al., 2014). Therefore, intracellular *Mtb* either can be eradicated through macrophage apoptosis (Lee et al., 2009) and autophagy (Alam et al., 2017), or can persistently survive and grow in macrophages. Indeed, *Mtb* can induce macrophage necrosis leading to the spread of infection to other cells by evolving an immune escape mechanism (Liu et al., 2017; Pajuelo et al., 2018; Zhai et al., 2019). Therefore, understanding the molecular mechanism of *Mtb*-induced macrophage deaths, particularly macrophage necrosis, may lead to uncover novel targets for host-directed therapy (HDT) of TB. Interestingly, ferroptosis is another form of programmed cell death (PCD) that is a type of necrosis dependent on iron (Dixon et al., 2012; Amaral et al., 2016). This PCD is induced by massive lipid peroxidation and has been demonstrated to be one of the mechanisms used by *Mtb* to spread after host infection. In the present study, Ma et al. investigated the role of HMOX1 in macrophage ferroptosis in response to mycobacterial infection by using transcriptome analysis of peripheral blood from TB patients. Moreover, the authors analysed HMOX1 mechanism in ferroptosis by employing mice and RAW264.7 cells infected with Bacillus Calmette-Guérin (BCG). Their results demonstrated that HO-1 is a negative regulator of murine macrophage ferroptosis during BCG infection, in part through a mechanism by which HO-1 inhibits intracellular ROS production and iron accumulation altering the death of *Mtb* in infected macrophages.

Yuan et al. studied the regulation of murine miR-25-3p, a miRNA that was upregulated on exosomes of macrophages after 72-h BCG infection. Initially, they determined by bioinformatics analysis that the target gene of miR-25-3p is *DUSP10* (dual-specificity protein phosphatase 10). Previously, Nomura et al. (2012) demonstrated the suppressive activity of DUSP10 on ERK (Nomura et al., 2012). Using a mimic and an inhibitor of mmu-miR-25-3p, a siRNA of DUSP10, and PD98059 (ERK Inhibitor) Yuan et al. evaluated the autophagy flux and the ability to eliminate BCG in murine macrophages. Thus, they demonstrated that mmu-miR-25-3 was able to inhibit DUSP10, promoting ERK1/2 phosphorylation and thereby increasing autophagy induced by BCG. Furthermore, they observed that upregulation of mmu-miR-25-3p also promotes autophagy and it was able to reduce BCG survival in Raw264.7 cells and facilitate the clearance of residual mycobacteria. Thus, miR-25-3p could be used for the construction of a novel anti-tuberculosis drug delivery system using exosomes as vectors loaded with antibiotics and specific miRNAs that have anti-TB and immunoregulatory functions.

Many biological and immunological aspects of TB are not completely elucidated, such as the complex process of immunoregulation mediated by regulatory T cells (Treg cells) and the enzymes indoleamine 2,3-dioxygenase (IDO) and heme oxygenase 1 (HO-1). In this study, Lozano-Ordaz et al. compared the contribution of these immunoregulatory factors in mice infected with *Mtb* strains with different levels of virulence: the mild virulence reference strain H37Rv or a highly virulent clinical isolate (strain 5186). The authors concluded that Treg cells, IDO and HO-1 activities are detrimental during late pulmonary TB induced by the mild virulence *Mtb*, probably because these factors decrease immune protection mediated by the Th1 response. In contrast, Treg cells, IDO and HO-1 are beneficial when the infection is produced by a highly virulent strain, possibly by regulation the excessive inflammation.

Osei-Wusu et al. used mycobacteria isolates including L4 (worldwide distributed) and L5 (West Africa-restricted and highly infectious) *Mtb* strains to study the association between bacterial and human genetics, particularly the inherent variation in the immune response of macrophages from Ghanaian Ewe and Akan self-reported ethnic groups. Data from *ex vivo* infections of monocytes-derived macrophages (MDMs) showed that Ewe MDMs exhibited a greater tendency to phagocyte L4 strains in comparison to Akan MDMs, while the latter displayed a higher replication rate for L4 *Mtb* strains. Conversely, Akan MDMs demonstrated increased uptake of L5, despite Ewe MDMs having a substantially higher replication rate for L5 strains. Moreover, the influence of self-reported ethnicity vanished when cells derived from the blood of cured TB cases were used. These observations corroborate findings from epidemiological studies where L5 infections were associated with the Ewe ethnic group (Asante-Poku et al., 2015). Thus, these findings suggest that host ethnicity (implying host genetic diversity), *Mtb* genetic diversity, and prior *Mtb* exposure collectively influence macrophage responses.

Another article in this Research Topic also explored the influence of host genetics and epigenetics on the susceptibility to *Mtb* infection, by affecting adaptive immune responses of TB patients. As it is well known, IFN-γ plays a key role in immune protection against *Mtb*. Single nucleotide polymorphisms in several candidate genes, especially polymorphisms in cytokine genes are known to modulate cytokine levels, which may influence susceptibility to tuberculosis infection and disease. Álvarez et al. propose of the +874 A/T (rs2430561) gene polymorphism, a genomic variant at a single nucleotide in the IFN-γ gene, as a possible genetic biomarker of susceptibility to TB. Specifically, demonstrated that AA genotype of the rs2430561 single nucleotide polymorphism was overrepresented in patients with active disease. They found that the A allele and AA genotype at rs2430561 are overrepresented in TB patients with active disease, and the presence of the T allele reduces IFN-γ production in *Mtb*-stimulated PBMCs in TB patients as compared to healthy donors. Furthermore, they observed increased DNA methylation levels in the IFN-γ promoter (in CpG island at position -53) in TB patients compared to individuals with latent TB. Therefore, methylation of this site could be used as a predictor of TB reactivation. is a genomic variant at a single base position in the DNA.

Immunometabolism has become a growing area of research with great impact on autoimmunity, cancer and infectious diseases. The metabolism of immune cells correlates with their microanatomical localization, activation, proliferation or function (O’Sullivan et al., 2019). By studying human CD1c+ myeloid dendritic cells from blood, Triglia et al. analysed glucose dependency of these cells when exposed to BCG, the only vaccine licensed to prevent TB disease. They showed that the challenge with BCG boosts the glycolytic function of CD1c+ mDCs, which has an impact in cytokine secretion and expression of CD40 and CCR7 but not in phagocytosis. Interestingly, this work showed that the maturation and migration of these cells in response to BCG are not exclusively dependent on the glycolytic pathway, and significant differences arise in this term between BCG-infected versus uninfected bystander DCs. These data highlight the complexity of the host response and provide evidence on the differences that can be found in immune responses to vaccines consisting of whole bacteria versus those composed of bacterial-derived antigens.

Exacerbation of the immune response is detrimental to host tissues and analysis of particular factors that counteract inflammation, such as corticosteroids and peroxisome proliferator-activated receptors (PPARs), is crucial. Accordingly, Diaz et al. investigated the contribution of PPAR Gamma to tuberculosis physiopathology. PPARs, which belong to the nuclear receptor superfamily, are a family of 3 ligand-activated transcription factors: PPARα (NR1C1), PPARβ/δ NR1C2), and PPARγ (NR1C3). These three subtypes of PPARs are encoded by different genes but show similar structural features. Diaz et al. showed that an increased expression of PPARsϒ in peripheral blood mononuclear cells (PBMCs) from patients was associated with TB severity. Moreover, by using THP-1 cells, the authors observed that radiation-killed *Mtb* modulated PPARsϒ expression and agonists decreased pro- and anti-inflammatory cytokines. As expected, cortisol treatment together with a PPARϒ agonist lowered the levels of this proinflammatory cytokine in stimulated cultures.

It has been described that people over 65 years old, face a notably elevated susceptibility and mortality to TB. This underscores that the elderly population constitutes a substantial reservoir for *Mtb* infection. In this Research Topic, Lafuse et al. analysed the effect of social disruption stress (SDR) on the lungs of young and old mice and their response to TB infection. This report shows that SDR induces the accumulation of CD11b+ myeloid cells in an age-independent manner but causes an immunosuppressive environment in the lung of aged mice characterized by reduced pro-inflammatory cytokine production (IL-1β, TNF and CXCL2) and augmented levels of norepinephrine. Interestingly, when challenged with *Mtb*, psychological stress in old mice negatively affects the control of the infection by increasing differentiation of IL-10-secreting T cells. Thus, this work demonstrates psycho-neuroimmune mechanisms that operate after psychological stress and might undermine disease susceptibility. Its relevance is manifested by considering that anxiety and depression are two common symptoms experienced by patients with TB (Wang et al., 2018; Duko et al., 2020), directly related with decreased treatment efficacy, physical skills, and reduced quality of life (Dos Santos et al., 2017; Ruiz-Grosso et al., 2020).

We acknowledge with gratitude the participation of reviewers and authors in this Research Topic, which allowed to achieve a collection of original and high impact articles that introduce new concepts in this field of research. TB remains a relentless threat, claiming the lives of thousands each year, with the most socially and economically disadvantaged populations paying the heaviest price. Continuous investment and research into pathogen interactions, host immune responses and variations in endotypes found in the population, will pave the way for better treatments, diagnostics and vaccines.

## Author contributions

JP: Conceptualization, Project administration, Writing – original draft, Writing – review & editing. MM: Project administration, Writing – original draft, Writing – review & editing. MC: Supervision, Writing – original draft, Writing – review & editing. VG: Conceptualization, Funding acquisition, Project administration, Supervision, Writing – original draft, Writing – review & editing.
